# How do physicians perceive quality of life? Ethical questioning in neonatology

**DOI:** 10.1186/s12910-015-0045-5

**Published:** 2015-07-24

**Authors:** Marie-Ange Einaudi, Catherine Gire, Pascal Auquier, Pierre Le Coz

**Affiliations:** UMR 7268 ADéS, Aix-Marseille University-EFS-CNRS, Espace Ethique Méditerranéen, Timone University Hospital, 13385 Marseille, France; Department of Neonatology, North University Hospital, Marseille, France; Aix-Marseille University, EA 3279 Self Perceived Health Assessment Research Unit, Marseille, France

**Keywords:** Autonomy, Ethics, Neonatology, Quality of life, Perception, Prematurity, Responsability

## Abstract

**Background:**

The outcome of very preterm infants is marked by the development of complications that can have an impact on the quality of life of the children and their families. The concept of quality of life and its evaluation in the long term raise semantic and ethical problems for French physicians in perinatal care. Our reflection aims to gain a better understanding of the representations surrounding quality of life in neonatal medicine.

**Discussion:**

If French physicians hesitate to face this concept (through self-interest and apprehension), it is because the debate has become more complex. Formerly, the dilemma was between respect for life versus quality of life. Today, although this dilemma is still with us, the questions raised by French physicians show us that autonomy is given increasing importance. The equation to be solved now contains three variables: respect for life, well-being, autonomy. So we find ourselves between three positions and no longer two: respect for life (the ethics of conviction), quality of life based on autonomy (rationalist and secular deontologism), and quality of life based on the differential between well-being and suffering (utilitarianism).

**Summary:**

A solution could lie in consequentialism, which integrates the consequences for future generations in terms of both safeguarding of autonomy and quality of life, and puts the sacredness of life in second place but without sacrificing it. By evaluating their future quality of life, we can better respond to the needs of these children.

## Background

Advances in perinatal medicine enable management of children who previously would not have survived, in particular extremely preterm infants, born before 28 weeks’ gestation. But, although this criterion is not exclusive, the lower the gestational age, the greater the mortality and morbidity [1–5].

Extremely preterm births pose decisional and ethical problems for the care teams: these dilemmas are mainly due to uncertainty as to the outcome of these children [6, 7]. The decision to undertake resuscitation at birth enables long-term survival without serious sequelae for the majority, but it may lead to the survival of children who sometimes have major disability. Conversely, the decision not to undertake resuscitation, in particular at the lowest gestational ages, implies accepting the death of infants who would perhaps have developed “normally” if they had been given care [6]. In general, the medical and psychosocial impact of the complications of extreme prematurity affects the daily life of these children and their families, with a non-negligible impact on their quality of life (QoL) [8].

Health-related QoL is an individual’s subjective perception of the consequences of their state of health on their physical, emotional and social development. As a criterion for evaluation of care strategies its use is still limited [9]. In the field of extreme prematurity, there are few reports on obstacles to its use.

Quality of life is a question that has not often been approached by French perinatal care physicians. We can wonder why. The issue at stake in this French ethical approach is to determine how French physicians concerned, perceive QoL. So, we propose a critical reflection on the concept of QoL in neonatal and perinatal medicine following upon research studies.

In the following, we will present briefly the main results of two French studies on the subject: the French preterm children QoL consideration and the physicians’ perception of such evaluation [10, 11]. Thereafter, on the basis of these results, we will explain in the discussion, how consideration of preterm children QoL could have its place in an ethical perspective.

### Taking quality of life into consideration

In the ethical reflection of French experts on perinatal care, QoL is discussed either from the angle of the child’s future QoL, or from that of the general impact on the family [6]. It is, however, approached through subjective reflection.

Whereas the QoL of children who were born preterm is well documented in the international literature, there are few French data on the subject [12]. A single study is available: it describes the QoL of 82 former preterm children of school age, which is a period of important issues in terms of learning, integration and awareness of disability [10]. The mean QoL scores reported by parents of preterm children were lower than those reported by parents of a reference French population. The factors involved in the QoL of these children were maternal parity, family socio-economic status and the presence of major neurocognitive disorders. This study raised two specific issues:

- the first issue concerns the small number of studies evaluating QoL in this age group (6–10 years), and this paucity may arise from a variety of factors [12]. The first factor, which is indisputable but non-specific, is that before the 1990s physicians were not attracted to this approach. This reflects not only lack of knowledge of the concept but also, on the one hand, distrust of a standardised approach and on the other the feeling by physicians that they were being dispossessed of their “own power” [13]. The second factor raises more specific and technical problems related to age. The child, in fact, considered as a moving target, raises the problem of the value of a single QoL questionnaire adapted for use over a very wide range of ages. Over the age of 8 years, the child may be considered as capable of replying, but at a younger age, the question arises of the “evaluator” who is best able to report on the child’s QoL. Even if the parents’ opinion appears the most pertinent, nevertheless that of the mother will not necessarily be the same as that of the father, and the question further arises of continuity of judgment between the parents’ opinion and that which will be reported by the children [14].
- the second issue concerns the apparent attenuation over the course of time of the effects of prematurity on QoL [15]. This apparent attenuation could make QoL a less relevant criterion, but it raises several questions with regard to evaluation methods (different tools for different age groups), differences in perception, coping ability, response shift, the patient’s environment, supportive measures and social and integration policies [9]. Overall, levels of QoL differ between countries and depend on the health system. It therefore seems difficult to limit our references only to the international literature.

The lack of available data and the limited use made of these data raise questions as to the place of QoL in the field of perinatal care and the perception that French physicians have of this qualitative aspect of health. It is what we are going to develop in the following.

### Physicians’ perception

In view of the paradoxes raised by technological progress and the dilemmas related to QoL, it seems interesting to approach the issue of QoL with expert physicians in perinatal care (the decision-makers) and with the physicians responsible for follow-up. What is their position with regard to evaluating QoL in extremely preterm children? We can already see that in line with the cultural changes of our society, QoL holds an increasingly important place [16]. What place could this concept have in the ethical reflection of physicians in perinatal care?

An opinion survey was carried out after ethical approval, in 78 French physicians (heads of obstetrics, neonatal medicine and paediatric neurology departments) by means of questionnaires developed after preliminary interviews: QoL was defined as a complex concept, as the physicians mainly took account of psychological and physical well-being and family relationships [11]. The concept was generally approached subjectively. The question was raised of its place in decisional ethics, or even the possibility of withholding or withdrawing treatment in view of diminished QoL. Indeed, while more than 88 % of physicians considered that the availability of data on quality of life would give new impetus to the ethical debate on neonatal resuscitation practices, nearly 90 % of physicians who responded to the survey, would consider therapeutic abstention if quality of life was compromised. Information on quality of life was considered more particularly useful in the neonatal period by 81 % of the physicians and in medium and long-term practice by 95 % of the physicians. Nevertheless, the physicians noted obstacles (methodological, conceptual, practical and ethical) to QoL evaluation; the mean being the difficulty face to the quantification of the qualitative. In spite of this, three main expectations were revealed: better understanding of needs, a better relationship with the patient, and, more unusually, a potential impact on decision-making (although some physicians considered such an impact as dangerous). Physicians are reticent towards the concept of QoL data and yet appear to seek information. Quality of life appears as an instrument that arouses both interest and apprehension. This opinion survey raises the question of the choice of target population (are physicians the members of the care team who are the most sensitive to the concept of QoL?) and it draws attention to the physicians’ perplexity with regard to this factor (explaining the fears or prejudices of the non-respondents).

In view of this observation, we set ourselves a double objective: to clarify the approach to QoL in extremely preterm infants from both a semantic and an ethical angle. We attempt to explain how, on the basis of the physicians’ declarations in the opinion survey, consideration of QoL can have its place in an ethical perspective. This reflection will be based on two types of approach: an approach based on the principles of biomedical ethics (applied with the educational aim of clarifying problems rather than solving them) and a more philosophical approach [17, 18].

## Discussion

Management of the extremely preterm newborn is marked by uncertainties of both prognosis and treatment. The outcome of some of these children is heavily compromised: suffering related to disability (severe disabilities as non ambulatory cerebral palsy, comprehensive retardation, severe visual or hearing deficiency…), repercussions on the family circle, social and economic family difficulties [19]. Doubt as to the outcome only increases the dilemmas over the decisions to be made [7]. In such a context, how can we introduce QoL considerations? What do they represent? What meaning is given to this factor? What are the ethical questions raised by physicians’ perception of QoL evaluation?

### Approach based on the principles of biomedical ethics

#### Dilemmas in perinatal medicine

The dilemmas that confront physicians in perinatal medicine call into question the principles of biomedical ethics [20]. Decisions are guided by the child’s best interests, a concept that oscillates between beneficence and non-maleficence [21]. Respect autonomy presupposes a capacity for intentional action on the basis of one’s own rational deliberations as well as freedom for controlling instances. So, the principle of autonomy is difficult to apply in this context. It does not apply to the newborn, nor does it wholly apply to his or her parents. Attempts must be made to collect their opinion, without making them bear the weight of the decisions, while making every effort to respect their own role in decision-making. The principle of justice is similar to a principle of equity, of just distribution of resources. It calls not only on the physicians but also on politics, and involves societal choices. This all takes place in a context of perinatal medicine where the issues at stake are high, to enable life or to permit death. This raises the classic value conflict between the sacredness of life and QoL.

#### Is QoL evaluation in harmony with these ethical principles?

French physicians seem reticent toward a quantitative evaluation of QoL, because they fear it may be a “reductive” tool [11]. Application of this concept thus appeared maleficent in view of the use to which it could be put (decisions to withhold or withdraw treatment), just like the idea that the value of life could be relative if it was evaluated in terms of quality. However, should we not make a distinction between the value of the person (absolute, non-measurable, unique) and their QoL (which can be evaluated)? In fact, are not QoL, the value of life, and the value of the person all different concepts?

This reflection raises a difficulty with regard to the norm. Paradoxically, measuring QoL is a matter of quantifying something that is qualitative. If we evaluate QoL and give it a quantitative value, this generally supposes that established norms exist. In fact, conformity to norms can define what is “normal”. Use of standards for QoL does not aim to define a “normal” life, but to take up a position in relation to reference scores, obtained in a general population and based on qualitative approaches [22]. Disability has first of all been subjectively experienced before it is measured [23]. But any norm implies reflection, whether the concept is objective or subjective. Is not the flexibility with which a norm can be used more important than its rigour? It is rather a question of considering these norms as having a regulatory function, indicating a direction, than of misusing them in a rigorist way.

In spite of these fears, taking QoL into consideration could bring back into the picture the autonomy of the children and the parents (who could express preferences and make decisions in the light of fuller, concrete information - supposing a ground for a right to self-determination and a right to be respected), with the aim of beneficence by fulfilling needs, improving patients’ well-being and reinvesting parents in their function. The constant preoccupation is to reduce potential suffering and so to respect the principle of non-maleficence. Ideally this supposes a more just distribution of resources, by reasserting the principle of long-term equity with regard to the needs of these children.

So the debate appears to become more complex: we are no longer only faced with the dilemma previously cited between sacredness of life and QoL. Although this dilemma is still with us, other parameters come into play, as we will attempt to develop in the following section.

### Philosophical approach

Faced by the dilemmas that arise in certain perinatal situations and the perplexity and hesitation of physicians over taking QoL into account in this particular context, it seems interesting to investigate this hesitation further. The question may be approached either on the basis of universal duties (a deontologist approach) or on the basis of the consequences (an utilitarianist approach), with neither perhaps taking precedence over the other.

To summarise the dilemma, physicians seem to be caught between two fears: the fear of using QoL as a value judgment on lives that are worth or not worth living, and the fear of potential suffering resulting from acts carried out in the neonatal period and having an impact on QoL. This dilemma brings face to face the essential values of the person, such as respect for life, and existential values such as autonomy or well-being which condition the individual’s QoL.

#### Deontologist arguments

In its classic form, the deontologist ethics lays the accent on the equal dignity of persons, while in its radical forms, it tends towards a form of the sacredness of life [24–26]. It leads to the formulation of reservations against a conception of QoL that could call into question respect for life. Quality of life would seem to depend on too many unknown quantities and unmeasurable parameters to be able to serve as a predominant criterion. Making distinctions between persons according to their QoL would lead to discrimination between lives that are worth living and those that are not. Taking into account the future QoL of extremely preterm infants could serve as a basis for eugenic decisions. The deontologist does not reason in terms of consequences but of universal duties, a viewpoint that comes close to an ethics of conviction, according to which nobody can decide on a person’s QoL [25, 26]. According to the rationalist, secular deontologist view, inspired by Kantian ethics, autonomy is intrinsically linked to our dignity as human beings and the absolute value of personhood [24, 27]. Autonomy is an unconditional value, which leaves us with a duty to remain autonomous. It is not any more the autonomy - principle described by Childress and Beauchamp, but a universal maxim, supposing honor an ideal of humanity in our acts, and respect the dignity in oneself and in others. So, autonomy seems important at the same time for the children and their parents.

#### Utilitarianist arguments

From an utilitarianist viewpoint, an action is good or useful if it improves the happiness of the greatest number referring to an hedonist approach of the utilitarianism [28, 29]. The impact of medical decisions could be examined in the light of the consequences and of the potential suffering. This is particularly important if the decisions have an impact on those close to the child, on the overall QoL of the patient and those close to the patient taken as a whole, and in particular that of the parents, closely linked to that of their child. This approach raises the question of the high costs incurred in the perinatal period by the management of these children and, overall, by the resources allocated to disabled persons. When the sum of wellbeing associated with care and treatment appears to be less than the amount of potential suffering, especially if it has an impact on the family circle, decisions to withhold or withdraw treatment could be taken. In this model taken to extremes, the idea of neonatal euthanasia could be considered: as the suffering of the family circle is real and tangible, it can be recognised and anticipated, while that of the newborn cannot [30]. But in this model of argumentation, it is inconceivable to justify euthanasia unless the newborn’s status as a person has been fundamentally contested…Utilitarianist reasoning can nevertheless be tempered by stressing the importance of respect for the dignity of the newborn and by responding to their needs, and it would authorise pro-active decisions depending on what is truly possible. A link between autonomy and QoL could be thought as something that benefits us and makes our lives better, approaching the notion of liberty [31]. The value of autonomy here could resemble the value that usually is ascribed to wellbeing or preference satisfaction (preferentialism) in consequentialist ethical theories.

#### Ethics of responsibility

How far neonatologists are prepared to go in order to save life where the prognosis is unclear ? How to decide? The question can be asked about the variations of perinatal practices [32]. The decision-making criteria depend on criteria forecasts, according to recommendations (as in France) or protocols of coverage but decisions should take themselves always individually, by granting a place to the parents will [1, 6]. Management of the extremely preterm infant gives rise to emotions of both compassion and fear, leading physicians to question their decisions, both in the perinatal period and during long-term follow-up. The basis of the question is the QoL that physicians foresee for children threatened with disability. Taking QoL into consideration means believing that anything that is done to the body is not done with impunity, without developmental, psychological, emotional or socioeconomic consequences. According to Hans Jonas, this responsibility in the face of the newborn’s frailty consists of asking what is good for the child, and it is the “heuristics of fear” which should arouse our awareness to the principle of responsibility for all that is frail and vulnerable [33]. All active responsibility is based on a preoccupation that could be expressed as follows: what will happen to the child if I do not take care of him or her? The question is all the more cogent because the infant’s survival depends on our acts.

Although they can often lead to opposing decisions, these two modes of argument (based on universal duties or consequences) may concur. It is our duty to respect patients’ dignity and autonomy, while maintaining a significant interest in the general good. Evaluation of QoL may respond to this aim, both from a deontologist viewpoint in terms of duty and obligation, and from a utilitarianist viewpoint in terms of consequences for the child and their family circle. Concern for the QoL of these children forms part of care, of the ethics of concern for the Other [26]. It means concern for the real life of the patient outside the medical sphere. Providing a response to their suffering means improving their well-being and so their QoL. Evaluation of QoL thus raises questions for physicians as to the field of their responsibilities, and obliges them to remain present to respond to the patients’ needs.

Consideration of the QoL of extremely preterm children is thus in favour of an ethics of responsibility, as described by Max Weber, challenging the dogmatic version of the ethics of conviction and giving more importance to weighting, an ethics of responsibility that accepts compromise [34]. The ethics of conviction and the ethics of responsibility are not contradictory, they are mutually complementary. In this sense, the choice not to resuscitate an extremely preterm newborn if his/her future QoL was found to be highly compromised (whether it is a decision *a priori*, withholding life-sustaining treatment, or a decision a posteriori, withdrawing life-sustaining treatment) would not be departing from values such as respect for their person, but a duty of responsibility, both by accepting the person in their own singularity, and also in this context of preterm births by respecting parental autonomy, a value in constant development [35]. Considerations regarding expected quality of life matter have been revealed in a larger opinion survey on treatment decisions regarding a newborn with severe brain damage [36]. The questioning is the same: how physicians should act and reason in such situations?

## Summary

The aim of this reflection was to achieve better understanding of the representations surrounding QoL in relation to extremely preterm births.

Whereas QoL appeared as a criticised concept (given the particular context of the perinatal period, with the possibility of medical termination of pregnancy, which in France can be performed up to term), reflection has made it possible to pass from preoccupation when faced with quantified evaluation of QoL, to preoccupation for the patient. The aim of QoL evaluation is not to stigmatise lives by designating them as good or bad. It cannot be reduced to a sacrificial use. It should be seen as an engagement to act, aiming to reduce the impact of the sequelae and to better support the families. In this way, it makes us focus again on the meaning and the consequences of our acts.

Change in perception of QoL raises questions as to the place currently given to the concept. In the light of ethical principles, one of the central semantic questions is whether QoL is a synonym of beneficence, or whether QoL should take account of potential autonomy, as the ideological and cultural changes in our society encourage us to do. In other terms, is the suffering of the child and its parents the only question to be raised, or should we include the child’s future capacity to participate in their own management? Our hypothesis is that QoL no longer relates only to the concepts of well-being or pleasure, but also takes into account the individual’s degree of independence. The increased value placed on autonomy in our society again raises the classic issue on the ethical level [37]. Two interpretations of the idea of autonomy seem valuable: autonomy conceived as a personal prudential value (something that benefits us in that it makes our lives better) or as an unconditional value in a Kantian sense [38]. Until recently, the dilemma was in fact formulated in terms of opposition between the utilitarianist vision and the personalist vision of man. The problem was to know whether one should respect life, its sacred nature, the dignity of the newborn, or give greater weight to the child’s QoL in the case of major disability. Henceforward, the goalposts have moved. There is a consensus on integrating the criterion of QoL in the decision, and disagreement centres on how it should be evaluated. The first position evaluates QoL according to the subject’s anticipated capacity to make free decisions [39]. The second position approaches the ethical problem at a collective level, looking at the survival of the extremely preterm infant from the angle of its qualitative impacts on the family unit, and beyond that, at its social repercussions.
